# The Angelina Jolie effect: how high celebrity profile can have a major impact on provision of cancer related services

**DOI:** 10.1186/s13058-014-0442-6

**Published:** 2014-09-19

**Authors:** D Gareth R Evans, Julian Barwell, Diana M Eccles, Amanda Collins, Louise Izatt, Chris Jacobs, Alan Donaldson, Angela F Brady, Andrew Cuthbert, Rachel Harrison, Sue Thomas, Anthony Howell, The FH02 Study Group, RGC teams, Zosia Miedzybrodzka, Alex Murray

**Affiliations:** 1 Genesis Breast Cancer Prevention Centre, University Hospital of South Manchester NHS Trust, Wythenshawe, Manchester, M23 9LT UK; 2 Manchester Centre for Genomic Medicine, Central Manchester Foundation Trust, St. Mary’s Hospital, Oxford Road, Manchester, M13 9WL UK; 3 Leicester Genetics Service, Leicester, LE1 5WW UK; 4 Regional Genetics Service Coxford Road Southampton, London, SO16 5YA UK; 5 Regional Genetics Service Guy’s Hospital, London, UK; 6 Regional Genetics Service, University Hospital Bristol, Clinical Genetics Unit, Bristol, St Michaels Hospital, Southwell Street, Bristol, BS28EG UK; 7 North West Thames Regional Genetics Service, Kennedy-Galton Centre, Watford Road, Harrow, Middlesex, HA1 3UJ UK; 8 Birmingham Women’s NHS Foundation Trust, Metchley Park Road, Edgbaston, Birmingham, B15 2TG UK; 9 Nottingham Regional Genetics Centre, Hucknall Road, Nottingham, NG5 1PB UK; 10 Nottingham University Hospital, Nottingham, UK; 11 University of Aberdeen Centre for Genome-enable Biology and Medicine, Ashgrove House, Foresterhill, Aberdeen, AB25 2ZA UK; 12 North of Scotland Regional Genetics Service, Ashgrove House, Foresterhill, Aberdeen, AB25 2ZA Scotland, UK; 13 Breast Test Wales and All Wales Genetics Service, Heath Park, Cardiff CARDIFF, CF14 4XW UK; 14 Genomic Medicine, MAHSC, St. Mary’s Hospital, Oxford Road, Manchester, M13 9WL UK

## Abstract

**Introduction:**

It is frequent for news items to lead to a short lived temporary increase in interest in a particular health related service, however it is rare for this to have a long lasting effect. In 2013, in the UK in particular, there has been unprecedented publicity in hereditary breast cancer, with Angelina Jolie’s decision to have genetic testing for the *BRCA1* gene and subsequently undergo risk reducing mastectomy (RRM), and a pre-release of the NICE guidelines on familial breast cancer in January and their final release on 26^th^ June. The release of NICE guidelines created a lot of publicity over the potential for use of chemoprevention using tamoxifen or raloxifene. However, the longest lasting news story was the release of details of film actress Angelina Jolie’s genetic test and surgery.

**Methods:**

To assess the potential effects of the ‘Angelina Jolie’ effect, referral data specific to breast cancer family history was obtained from around the UK for the years 2012 and 2013. A consortium of over 30 breast cancer family history clinics that have contributed to two research studies on early breast surveillance were asked to participate as well as 10 genetics centres. Monthly referrals to each service were collated and increases from 2012 to 2013 assessed.

**Results:**

Data from 12 family history clinics and 9 regional genetics services showed a rise in referrals from May 2013 onwards. Referrals were nearly 2.5 fold in June and July 2013 from 1,981 (2012) to 4,847 (2013) and remained at around two-fold to October 2013. Demand for *BRCA1/2* testing almost doubled and there were also many more enquiries for risk reducing mastectomy. Internal review shows that there was no increase in inappropriate referrals.

**Conclusions:**

The Angelina Jolie effect has been long lasting and global, and appears to have increased referrals to centres appropriately.

**Electronic supplementary material:**

The online version of this article (doi:10.1186/s13058-014-0442-6) contains supplementary material, which is available to authorized users.

## Introduction

It is frequent for news items to lead to a short-lived temporary increase in interest in a particular health-related service. It is rare for this to have a long-lasting effect. In 2013 in the UK in particular there has been unprecedented publicity on hereditary breast cancer with a pre-release of the National Institute of Health and Care Excellence (NICE) guidelines on familial breast cancer in January 2013 and their final release on 26 June. The release of NICE guidelines created much publicity over the potential for use of chemoprevention [1] using tamoxifen or raloxifene. However, the longest-lasting news story was the release of details surrounding the film actress Angelina Jolie’s decision to have genetic testing for the *BRCA1* gene and subsequently undergo risk-reducing mastectomy (RRM). Unusually this story lasted several weeks in the newspapers and on TV and radio, and longer in magazines. Indeed the story resurfaced with news items on the BBC in December. The so-called Angelina Jolie effect prompted publicity across the English-speaking world with articles in newspapers about the effects on breast screening, hereditary breast cancer clinics and genetics services in the USA, Canada, Australia, New Zealand and the UK [2]-[6]. An article in Australia confirmed a tripling in breast cancer referrals for genetics, family cancer centres in Victoria, New South Wales and South Australia, which had a combined 90 referrals a week in the six weeks before Ms Jolie’s announcement, which doubled and then tripled to a peak of 280 referrals in the weeks after. The article from November some 6 months after the Jolie story broke on 14 May, showed that referrals had since settled at about 190 a week, demonstrating the Angelina effect was ongoing. There was some initial press criticism of Ms Jolie’s decision to undergo RRM, but this abated to a large extent when it was announced 15 days after the initial news story that her aunt had died of breast cancer (the initial stories only mentioned ovarian cancer in her mother). Calls to the Hereditary Breast Cancer Helpline in the UK have increased 10-fold and still remained high in January 2014 [7]. The helpline states that no other news story has had such an effect [7]. All the familial breast cancer clinics we have contacted in the UK have noted increases in referrals that they attribute to the story.

### Familial breast cancer services

**Figure 1:**
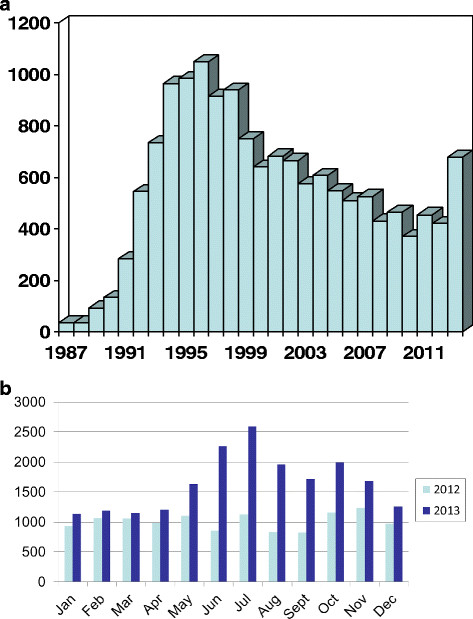
**Breast cancer family history (FH) referrals. (a)** Breast cancer FH referrals to the South Manchester family history clinic (FHC) showing exponential rise in referrals in early 1990s and second rise in 2013. **(b)** Breast cancer FH referrals to 21 centres in 2012/2013 by month. 12 FHCs and 9 UK Genetic services.

Breast cancer family history clinics (FHCs) have existed in the UK since 1986 [8],[9]. Each service saw an initial exponential rise in referrals (Figure 1a), which was to some extent caused by a new service meeting an unfulfilled need. Most services then saw a plateau in referrals. In the UK referrals of women with a family history of breast cancer have largely been triaged since the mid 1990s with average-risk women being reassured in primary care and moderate-risk women aged <50 years of age potentially gaining access to additional surveillance mammography at local FHCs [10], with those at high risk (>1 in 4 lifetime risk) gaining access to genetics services and potentially testing for *BRCA1/2* mutations [10]. These guidelines were eventually enshrined into NICE guidance with the publication of the first guideline on familial breast cancer in 2004 [11]. The most recent guidance was summarised in this journal recently and reduced the threshold for genetic testing for *BRCA1/2* mutations to 10% [12].

## Methods

### Assessment of the Angelina Jolie effect

To assess the potential effects of the Angelina Jolie effect, referral data specific to breast cancer family history was obtained from around the UK for the years 2012 and 2013. A consortium of 34 breast cancer FHCs that have contributed to two research studies on early breast surveillance [13],[14] were asked to participate. Likewise 10/19 of the Regional Genetics Centres (RGCs) that were approached had cancer-specific referral data (one of the centres could not separate breast from other cancers) and were invited (Table 1). Monthly referrals to each service were collated and increases from 2012 to 2013 assessed. In Manchester the appropriateness of referrals against local guidelines was assessed. This study was an audit and did not require ethical approval.

**Table 1:** **National Health Service Regional Genetics Centres and family history clinics, their potential catchment area and referrals in 2012/2013**

Centre	Type	Population coverage	Number referred 2012	Number referred 2013
Guys Hospital, SW Thames, London	Regional Genetics Centre	4.9 million	1,762	2,727
Birmingham	Regional Genetics Centre	5.5 million	1,993	3,421
Southampton	Regional Genetics Centre	Approximately 3 million	735	1,032
Leicester	Regional Genetics Centre	Approximately 2 million	331	443
Aberdeen, Scotland	Regional Genetics Centre	Approximately 1 million	387	742
Bristol	Regional Genetics Centre	2.46 million	919	1,462
All Wales Genetics Service	National Genetics Centre	3.1 million	1,462	2,727
Nottingham	Regional Genetics Centre	2.2 million	1,015	1,252
Northwick Park, London	Regional Genetics Centre	3.6 million	760	1,902
Genesis Prevention Centre, Manchester	Family history clinic	4.5 million (for high risk)	367	678
Royal Marsden, London	Family history clinic	<1 million	255	320
Nottingham	Family history clinic	~1 million	554	739
Bath	Family history clinic	<1 million	166	278
St Bartholomew’s, London	Family history clinic	<1 million	538	627
Royal Derby Hospital	Family history clinic	<1 million	285	511
United Lincolnshire Hospitals NHS Trust	Family history clinic	<0.5 million	33	53
Sandwell Hospital, Birmingham	Family history clinic	<1 million	78	48
Edinburgh, Scotland	Family history clinic	<1 million	73	160
Leighton Hospital, Crewe	Family history clinic	<1 million	121	172
Coventry	Family history clinic	<0.5 million	178	192
Altnagelvin Hospital, N Ireland	Family history clinic	<1 million	130	202

## Results

Referral patterns for the 21 centres (12 FHCs and 9 RGCs) that participated are shown in Figure 1b and Table 1. Centres that did not supply data either did not have this available or were unable to collate the data. The data were available across Wales through the All Wales Genetics Service, covering seven Welsh regions and a population of around 3.1 million people. Further data were available from the Genesis Prevention Centre in Manchester and FHCs in Crewe, Bath, The Royal Marsden and St Bartholomew’s London, Derby, Coventry, Nottingham, Grantham, City Hospital Birmingham, Londonderry and Edinburgh. RGC data were available from Aberdeen, Leicester, Bristol, Guy’s and North-West Thames, London, Nottingham, Birmingham and Southampton.

Although referral rates were 17% higher in January to April 2013, the rates clearly rose further in May and June before an effect from the release of the full NICE guidance would have been seen. The nearly 50% increase in May reflects that only half the month would have been available to women to see their general practitioners (GPs) for a referral. Rates were around 2.5-fold higher in June and July from 1,981 (2012) to 4,847 (2013) and around 2-fold higher still in August through to October. The referral rates then settled back to 32% higher in November/December.

The extensive publicity when the updated NICE clinical guideline was published focused largely on chemoprevention and resulted in only a marginal increase in referrals relating to use of tamoxifen or raloxifene. In contrast, all participating centres were conscious of a more significant increase in women attending referring to the Angelina Jolie story and further, noted women seen in the past seeking updated advice on testing and risk-reducing surgery. Areas with very strong FHCs saw less pronounced effects in their RGCs. Altogether referrals rose from 12,142 in 2012 to 19,751 in 2013. Data from the Manchester FHC showed that 451/678 (Figure 1b, 66.5%) of referrals in 2013 were eligible to be seen after a questionnaire was received compared to similar proportions in the two previous years 265/421 (63%) in 2012 and 304/455 (67%) in 2011. The effects on referrals for genetic testing are likely to be confounded by the change in NICE guidance threshold that was heralded in January 2013 and ratified in June. Nonetheless, in seven RGCs (Manchester, Leicester, Nottingham, Wales, Southampton, NW Thames, Guys, East of Scotland) we saw a rise from 538 full *BRCA1/2* tests in July 2012 to December 2012 compared to 967 (80% increase) for the same period in 2013, despite no extra funding being available for testing. The effects on referral for RRM were also assessable in Manchester for the same 6-month periods. As it takes an average of 8 months for women to go through two genetic counselling sessions a psychological assessment and two surgical sessions before surgery is possible in women unaffected with breast cancer [12],[14], we assessed the rates of referral for psychological assessment in the same study periods (July to December). There were 13 referrals in 2012 compared to 24 in 2013. All services have found the increase in demand through referrals and increased genetic testing difficult as there is no mechanism for additional funding in the UK Health service to cover such eventualities.

### Controls

We were also able to assess familial colorectal cancer or other non-breast cancer referrals for the same two-year period from six RGCs as a comparison. There was no substantial rise from 2012 to 2013 and in particular no trend around the Jolie news story or NICE guidance on familial breast cancer.

## Discussion

### Publications of the Angelina Jolie effect

A survey carried out in the USA [15] found that although 75% of Americans were aware of Angelina Jolie’s double mastectomy, fewer than 10% of respondents had the information necessary to accurately interpret her risk of developing cancer relative to a woman unaffected by the BRCA gene mutation. Awareness of the Angelina Jolie story was not associated with improved understanding. However, 9% of women were motivated to do something about their health, such as seeing a doctor, having a mammogram or seeing a genetic counselor. The increased level of appropriate referrals in the UK may reflect a similar effect. The authors concluded that although celebrities can bring heightened awareness to health issues, there is a need for these messages to be accompanied by more purposeful communication efforts to assist the public understanding. There is no evidence from the current study that the story led to inappropriate referrals although it is possible that primary care physicians had to see many women who were unnecessarily worried about breast cancer, or observed an increased need for BRCA testing in the months following the revelation. It is likely that the release of NICE guidance with the attendant publicity may have made it easier to deal with the onslaught of enquiries about familial breast cancer. It is also likely that given the high level of appropriate referrals received in clinics, that the triage process set up in the 1990s is still effective today. Nevertheless, the high number of appropriate referrals means that many women will have been either unaware of the relevance of their family history or hiding concerns such that so many newly identified women and families could come forward in a 7-month period.

### Similar stories

A similar effect on health service activity happened six years ago, when a reality TV star Jade Goody was diagnosed with and then died of cervical cancer [16]. There are parallels in the media coverage around this UK story as there were multiple news items over several months. A study, in the Journal of Medical Screening, discussed the effect of her diagnosis and death on cervical screening attendance [17]. It showed that more than 400,000 extra women were screened in England between mid 2008 and mid 2009 - the period during which Jade Goody was diagnosed with and died of cervical cancer.

More women of all ages were screened, though the increase was greater for women aged <50 years. In the 25 to 29 years age-group, an estimated 31,000 extra women were screened in 5 months between autumn 2008 and spring 2009. It appeared that women closest to Jade Goody’s age or circumstances were those most affected by her experience [18]. Data from 890 participants showed that 40% of women felt Goody’s story had influenced their decisions about cervical screening. Younger women (aged 26 to 35 years) were more likely to have been influenced by Goody’s story than older women [18].

A similar trend was seen in bowel cancer screening in the USA after Katie Couric’s colorectal cancer awareness campaign on colonoscopy rates on the Today Show in March 2000 [19]. The number of colonoscopies performed per month after Ms Couric’s campaign increased significantly (15.0 per month before and 18.1 per month after the campaign; *P* <0.001). After adjusting for temporal trends, a significantly higher post-campaign colonoscopy rate was sustained for 9 months [19].

Although there was concern that the increase in attendance might have been from the so-called worried-well coming back for an early repeat screen, the research found that the opposite was true. A higher proportion was from women who were late for their test, rather than those who were coming back early [18]. In the 25 to 49 years age group, for example, 82,000 (28%) women had not been tested for five years or longer, while only 7,500 (8%) were coming back early, having already been screened in the past three years. The increase in appropriate use of health service resources from the Jade Goody effect appears similar to that of the Angelina Jolie effect. Other examples of notable women increasing or changing the use of health resources include a 40% increase in breast screening in Australia with the news around Kylie Minogue’s diagnosis [20], and a 6-month 25% increase in mastectomy for breast cancer after Nancy Reagan’s decision not to have breast-conserving surgery in 1987 [21]. All these stories, including the current one, show that health news around high profile individuals can have a sustained effect for at least 6 months in influencing the uptake of healthcare. We have shown that for Angelina Jolie the effect has been UK-wide as well as the reported global effects [2]-[7]. The increased awareness of familial cancer in the community alongside improvements in genetic testing, screening and preventative strategies, provides funding challenges for clinical genetic services and commissioners.

There are some limitations in the descriptive nature of the present study and ideally a prospective study immediately investigating motivations for referral would likely have added support to the findings. Future studies might anticipate a celebrity providing publicity in a healthcare area and design studies to gain a more in-depth understanding of referral patterns.

## Conclusions

Angelina Jolie stating she has a *BRCA1* mutation and going on to have a RRM is likely to have had a bigger impact than other celebrity announcements possibly due to her glamorous image and relationship to Brad Pitt. This may have lessened patients’ fears about a loss of sexual identity post preventative surgery and encouraged those who had not previously engaged with health services to consider genetic testing. It is not currently standard practice to proactively take a family history of cancer in primary care [11]. Hence there is an onus on at-risk relatives to be aware of their family history and request screening or risk-reducing strategies, resulting in possible inequality of access. Education of the general public is therefore extremely important in increasing awareness of, and improving access to, familial cancer services. This is particularly relevant due to the NICE guidance update increasing access to genetic testing, screening and chemoprevention [12].

## Authors’ original submitted files for images

Below are the links to the authors’ original submitted files for images. Authors’ original file for figure 1

## Abbreviations

BBC: British Broadcasting Corporation
FHCs: family history clinics
NICE: National Institute of Health and Care Excellence (England and Wales)
RGCs: Regional Genetics Centres
RRM: risk reducing mastectomy
