# Supplementary figures and images for: The Angelina Jolie effect: how high celebrity profile can have a major impact on provision of cancer related services
Source: Breast Cancer Res. 2014 Sep 19;16:442. doi: 10.1186/s13058-014-0442-6 (PMC4303122; doi:10.1186/s13058-014-0442-6)

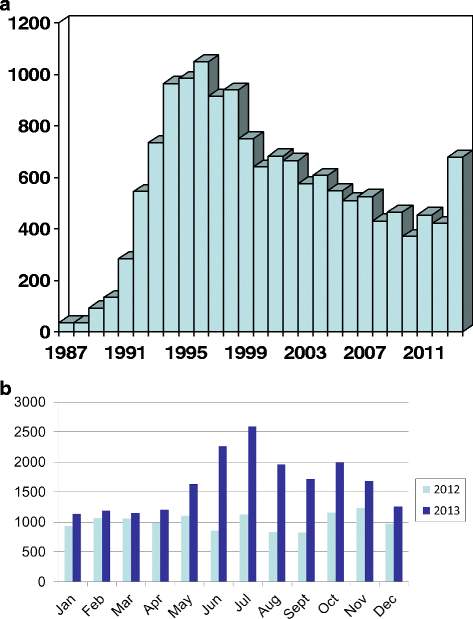

Supplement: Supplementary file 1 — Authors’ original file for figure 1 [file 13058_2014_442_MOESM1_ESM.gif]
